# Paths for colonization or exodus? New insights from the brown bear (*Ursus arctos*) population of the Cantabrian Mountains

**DOI:** 10.1371/journal.pone.0227302

**Published:** 2020-01-31

**Authors:** Inês Gregório, Tânia Barros, Doriana Pando, Joaquín Morante, Carlos Fonseca, Eduardo Ferreira

**Affiliations:** 1 Department of Biology & CESAM, University of Aveiro, Campus Universitário de Santiago, Aveiro, Portugal; 2 Fondo para la Protección de los Animales Salvajes, Santo Adriano, Asturias, Spain; University of Warsaw, POLAND

## Abstract

Over the centuries, the geographical distribution of brown bear (*Ursus arctos*) across the Iberian Peninsula has been decreasing, with the species currently confined to North Iberia. The Cantabrian brown bear population is one of the smallest in Europe and is structured into two subpopulations, positioned along an east-west axis. Given the current critically endangered status of this population, it is essential to have a clear picture of its within-population genetic patterns and processes. We use a set of three molecular markers (mitochondrial DNA, autosomal microsatellites and sex markers) to clarify the genetic origins and assess the migration patterns and gene flow of the Cantabrian brown bear population. Our results reveal the presence of two different mitochondrial (matrilineal) haplotypes in the Cantabrian population, both belonging to European brown bear clade 1a. The two haplotypes are geographically structured between Eastern (haplotype CanE) and Western Cantabrian (haplotype CanW) subpopulations, which is consistent with the genetic structure previously identified using nuclear markers. Additionally, we show that CanE is closer to the historical Pyrenean (Pyr) haplotype than to CanW. Despite strong structuring at the levels of mtDNA and nuclear loci, there is evidence of bidirectional gene flow and admixture among subpopulations. Gene flow is asymmetrical and significantly more intense from the Eastern to the Western Cantabrian subpopulation. In fact, we only detected first generation male migrants from the Eastern to the Western Cantabrian subpopulation. These results suggest more intense migration from the smaller and more vulnerable Eastern Cantabrian subpopulation towards the larger and more stable Western Cantabrian subpopulation. These new insights are relevant for assessments of on-going conservation measures, namely the role of dispersal corridors and enhanced connectivity. We discuss the importance of complementary conservation measures, such as human-wildlife conflict mitigation and habitat improvement, for the conservation of a viable Cantabrian brown bear population.

## Introduction

Large carnivores are one of the most challenging groups of species to conserve due to their large territories, broad dispersal ranges, low densities and reproduction rates, direct persecution by humans and due to other factors concerning human-wildlife conflict [1,2]. Together, these factors increase their vulnerability to extinction. Moreover, small isolated populations of carnivores often present low genetic diversity, which can translate into lower adaptability and survival when faced with environmental change [3,4,5].

The global population of the brown bear *Ursus arctos* is widely distributed and stable [6]. However, the southern range of this species primarily consists of small and fragmented populations that are locally endangered, as is the case for the Cantabrian brown bear population, which represents one of the smallest populations in Europe. This population is fragmented across a mountain range into two subpopulations (Western and Eastern) separated by 50 km [7,8]. Human persecution and poaching represent serious threats to the Cantabrian brown bear population, especially for the Eastern subpopulation [9]. Furthermore, connectivity between both subpopulations is limited, resulting in isolation and, consequently, reduced connectivity and gene flow [10,11,12]. Considering the current conservation status of the Cantabrian brown bear population, it is important to understand contemporary genetic patterns in both subpopulations in order to inform conservation and management strategies and ensure their long-term survival.

To assess the genetic structure and diversity of the Cantabrian brown bear, we had four principle goals in this study. First, we wanted to establish the origins and phylogeographic affinities of the Cantabrian brown bear. Two main mitochondrial DNA lineages (Western, divided in clades 1a and 1b; and Eastern, which includes clade 3a) of brown bear are known to occur in Europe [13,14,15], but the phylogeographic affinities between the two Cantabrian subpopulations and their relationships to other Iberian and European populations are unknown. Secondly, we aimed to link Cantabrian brown bear genetic structure detected using nuclear markers with patterns detected using matrilineal (mitochondrial DNA, mtDNA) markers. Genetic structuring would help us understand population dynamics and constitute a basis for answering other questions related to migration, gene flow and sex-biased dispersal [16,17]. Thirdly, we endeavoured to evaluate the genetic health of the Cantabrian brown bear population by estimating effective population sizes (N_e_), levels of endogamy, and by detecting genetic bottlenecks, all parameters that can influence the genetic diversity and thus genetic health of a population. Finally, given that connectivity and gene flow contribute to preventing inbreeding and can promote genetic diversity within a population [16,18], we assessed the degree of connectivity between the two Cantabrian brown bear subpopulations. We believe that the outcomes of this study provide a broader picture of the genetic condition, health and population dynamics of the brown bear population in the Cantabrian Mountains.

## Materials and methods

### Study area

**Fig 1:**
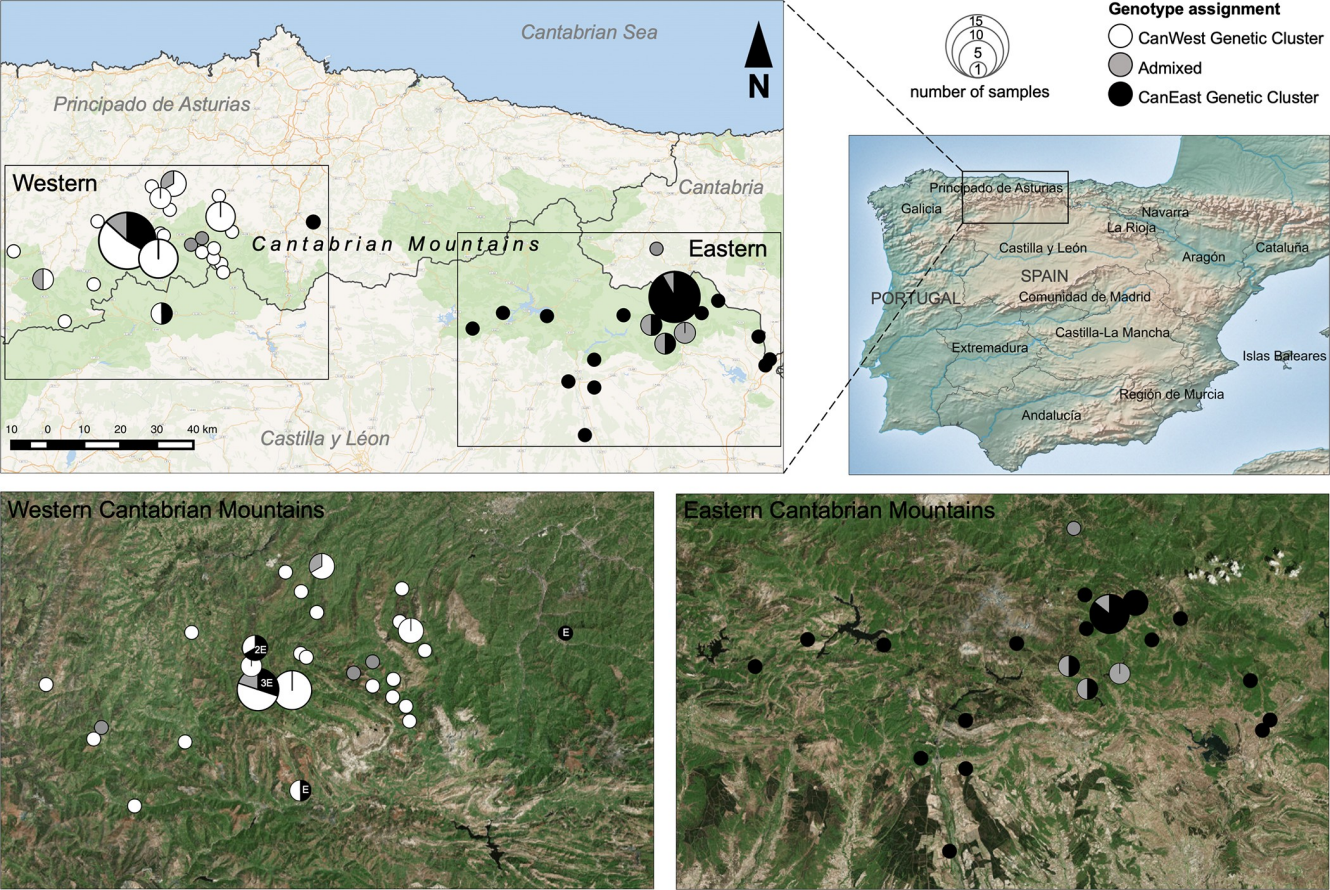
Map of the study area, with sampling locations for all genotyped individuals. Individuals are identified according to their assignment to genetic clusters or their admixed origin (based on STRUCTURE results and the thresholds inferred in HYBRIDLABS): CanWest (white); CanEast (black); admixed (grey). All individuals sampled from the Eastern Cantabrian population present the CanE haplotype and all individuals sampled from the Western Cantabrian population present the CanW haplotype, apart from seven individuals denoted “E”. Maps modified from: Natural Earth (naturalearthdata.com) physical map (top right); satellite images from Copernicus Sentinel Data (2017) for Sentinel-2A/B, obtained from the Copernicus open access hub (bottom); data from OpenStreetMap contributors, through Wikimedia Commons QGIS unlabelled layer (top left).

This study was conducted in the Cantabrian Mountains, located along the Atlantic coast of north-western Spain, spanning the provinces of Asturias, Cantabria, León, Lugo and Palencia (Fig 1). The Cantabrian Mountains display considerable geomorphological heterogeneity and have a complex topography, with altitudes ranging from sea level to 2647 m [19,20]. The northern (Atlantic) slopes of the mountain range are characterized by steep and narrow valleys, with abundant precipitation and humidity, whereas the southern slopes have wider valleys and precipitation occurs mainly during winter. Given its characteristics, the mountain range marks the transition between the Euro-Siberian and Mediterranean phylogeographic regions [21]. Forest coverage represents about 25% of the total area and is mainly characterized by beech (*Fagus sylvatica*), oaks (*Quercus pyrenaica*, *Quercus petraea*, *Quercus ilex*), birch (*Betula alba*), holly (*Ilex aquifolium*), chestnut (*Castanea sativa*) and hazel (*Corylus avellana*). The landscape is mostly characterized by shrubland (*Juniperus communis*, *Vaccinium uliginosum*, *Vaccinium myrtillus*, *Arctostaphylos uva-ursi*) at altitudes higher than 1700 m [19,22]. Although the human population density in the area is low, human activities have converted large patches of natural cover into pastures and agricultural lands. This habitat conversion has resulted in highly fragmented forested areas [19] and less suitable habitat for brown bears, increasing their vulnerability.

### Sample collection and DNA extraction

A total of 142 samples were collected by experienced field technicians from *Fondo para la Protección de los Animales Salvajes* (FAPAS) between 2010 and 2017, under the framework agreement signed between the Regional Administration and FAPAS for the application of the Brown Bear Recovery Plan. Most samples were collected between 2016 and 2017 (83%), and consisted of hair samples collected from fences, barbed wire or tree bark along known brown bear paths. Hair-traps, monitored by camera-traps, were also used to individually sample brown bears. Four muscle samples were kindly provided by Principado de Asturias, which had been collected from bear carcasses found in the field. Tissue samples were stored in 70% ethanol and hair samples were dried and preserved in individual paper envelopes at room temperature and in a dry environment until further analysis. DNA extraction was conducted using QIAGEN® *DNeasy Blood and Tissue Kit*, following the manufacturer’s instructions.

### Mitochondrial DNA amplification and sequencing

A 269 base pair (bp) fragment of the mtDNA control region was amplified using the primers developed by Taberlet and Bouvet [14]. PCR amplification was performed using INVITROGEN^®^ *Taq* DNA Polymerase and following the manufacturer’s conditions, with an annealing temperature of 50 ºC. PCR products were purified and sequenced using an ABIPRISM^®^ 3730-XL DNA Analyser from Applied Biosystems^™^. Sequences were aligned using MEGA 7.0 [23] with the CLUSTALW algorithm [24], and alignments were manually edited afterwards.

### Microsatellite amplification and genotyping

A total of 15 autosomal and two sex markers were amplified in four multiplex sets, comprising five (MU50, MU23, MU59, G10L, SRY), six (G10P, G10J, G1A, MU61, MU51, AMLX/Y) and three (G10X, G1D, MU05; G10C, MU09, MU10) loci [3,25,26,27,28]. DNA amplifications were performed using the QIAGEN® *Multiplex amplification kit* and following the manufacturer’s conditions, with an annealing temperature of 57 ºC. PCR products were analysed using an ABIPRISM® 3730-XL DNA Analyser. In order to reduce the likelihood of mistyping errors, each sample was independently amplified and genotyped a minimum of three times for each locus. Microsatellite genotyping was performed using GENEMARKER^™^ v2.4.1 [29]. Allele calling was performed manually, with careful inspection of electrophoretograms. Individual profiles (S1 Table) were inferred only when at least 12 microsatellite markers were successfully amplified (in most cases, we successfully genotyped at 14 or more markers).

### Data analyses

#### Phylogeographic affinities

To infer phylogeographic affinities of the Cantabrian brown bear, we retrieved 81 mtDNA control region haplotypes of Eurasian bears from GenBank (see details and references in S2 Table) and included them in an analysis with the haplotypes we obtained in this study. Three sequences from Asia and North America were used as outgroups for Bayesian inference. Numbers of individuals per haplotype were obtained from the original publications. We divided the haplotypes according to geographic origin, i.e. Iberian Peninsula, Apennine Peninsula, Balkan Peninsula, Carpathians, Scandinavia, Middle East, or NW Russia, Baltic & Finland.

Evolutionary pathways among the different haplotypes were assessed through a haplotype network generated in POPART 1.7 [30] using a median-joining algorithm [31]. The median-joining network was constructed with equal weights for all mutations and setting the parameter *ɛ* to zero to restrict the choice of feasible links in the final network. Phylogenetic relationships among haplotypes were inferred using a Bayesian approach. The Hasegawa-Kishino–Yano (HKY) model of nucleotide substitution represented the best-fitting model and was selected using MrMODELTEST 2.3 [32]. Inferred parameters were used as priors in MrBAYES 3.2 [33]. Two independent runs of four Markov chain Monte Carlo (MCMC) permutations were performed for 1,500,000 generations, sampling every 100 generations. Consensus trees (50%) were drawn using FIGTREE 1.4.0 [34] after discarding the first 25% of iterations as burn-in.

#### Genetic patterns and structure units

MICROCHECKER 2.2.3 [35] was used to test the final marker dataset for potential errors and matches between different samples were identified using GENALEX 6.5 [36]. Since evidence for null alleles or allele dropout can be biased by population structure and admixture, we tested the Eastern and Western Cantabrian populations separately. Probability of identity (P_ID(SIBS)_) was estimated in GENALEX 6.5 for a minimum of 12 loci, using a conservative method that assumes a population of siblings [37]. When matches between two different samples were detected, we deemed one of the samples to be a recapture and removed it from the dataset. All 15 loci were tested for deviations from Hardy-Weinberg equilibrium (HWE) and linkage equilibrium (LE) in ARLEQUIN 3.5.1.2 [38]. Bonferroni corrections were applied for all multiple comparisons. We searched for evidence of genetic structure using STRUCTURE 2.3.4 [39], applying an admixture model with correlated allele frequencies and no prior information about the original population of each individual. We ran 10 replicate runs of the analysis, for 2,000,000 MCMC iterations, with a burn-in of 100,000 steps and with K number of populations ranging from 1 to 6. We used STRUCTURE HARVESTER [40] to summarize the results and estimated the best K using the Evanno method [41].

To formally assess partitioning of genetic variation among the identified subpopulations, we performed a standard analysis of molecular variance (AMOVA). The significance of the inferred genetic structure was assessed through pairwise F_ST_ [42]. These analyses were performed with 10,000 permutations in ARLEQUIN.

#### Genetic and demographic parameters

We estimated the number of alleles (N_A_), rarefied allelic richness (A_r_), observed (H_O_) and expected (H_E_) heterozygosities, and the inbreeding coefficient (F_IS_) using ARLEQUIN. Evidence of bottlenecks for each inferred cluster was tested using MRATIO [43] and BOTTLENECK 1.2.02 [44]. In MRATIO, we used a set of conservative parameter values for simulations, with Δg = 3.5 (Δg: mean size of larger mutations) and *ps* = 0.9 (*ps*: mean % of mutations that add or delete only one repeat) [43]. The parameter Θ was allowed to vary over several orders of magnitude (0.01, 0.1, 1 and 5) to account for a wide range of mutation rates and pre-bottleneck effective population sizes. In BOTTLENECK, simulations were performed using a two-phased model (T.P.M), with 70% S.M.M., 20% variance and 1,000 replicates. Wilcoxon sign-rank tests were applied to determine the significance of heterozygosity excess or deficiency for each model.

We assessed the effective population size (N_e_) using the linkage disequilibrium method [45], whereas the effective number of breeders (N_eb_) was determined using the molecular co-ancestry method [46]. Both methods were implemented in NeESTIMATOR v2 [47]. The 95% confidence intervals for both methods were obtained through a Jackknife method and estimates for the linkage disequilibrium method excluded all alleles with a frequency <0.05 to correct for known biases from rare alleles.

#### Connectivity and gene flow between subpopulations

We calculated the likelihood of assignment of individual genotypes to both subpopulations with GENALEX. To assess the most conservative *Q* posterior probability of assignment to parental subpopulations and the potential of the studied loci to detect admixed individuals, we tested the power of the Bayesian-based STRUCTURE method by performing simulations in HYBRIDLAB 1.0 [48]. Multilocus genotypes were simulated by sampling alleles from the inferred populations shown in STRUCTURE, assuming random mating, linkage equilibrium and neutrality. Since we determined the most likely K for the Cantabrian bear population to be K = 2 based on the Evanno method, we selected 15 individuals from each of the two inferred subpopulations using a Tq threshold value of Tq>0.90 [39]. We then simulated a total of 100 genotypes for each of the admixed classes: F1, F2, and backcrosses BxOa and BxOb. Assignment tests for simulated genotypes were performed under the same conditions as for the real dataset in STRUCTURE. First generation migrants were identified by combining the results from STRUCTURE and HYBRIDLABS with those obtained using BAYESASS 3.0.4 [49] and GENECLASS 2.0 [50]. In BAYESASS, the MCMC algorithm was run for 10,000,000 iterations (three replicates), with a burn-in period of 1,000,000 and a sampling frequency of 1000. In GENECLASS, migrant detection was performed using the algorithm developed by Paetkau and collaborators [51].

We estimated the degree and direction of asymmetric gene flow among subpopulations using the relative migration network method developed by Sundqvist et al. [52], which is implemented in the function *divMigrate* of the diveRsity R package [53]. A significant relative migration network was estimated based on a bootstrap procedure with 50,000 replicates. Estimates of migration rates obtained using this method (which relies on differences in allele frequencies) were compared with the recent migration rates obtained using BAYESASS (which are inferred from genotypes).

## Results

Our final dataset included 80 unique genotypes, corresponding to 50 and 30 bears captured from the Western and Eastern Cantabrian subpopulations, respectively. The probability of identity, assuming a population of siblings, for the whole Cantabrian population was 7.0x10^-4^ or 1.1x10^-4^ considering 12 or all 15 loci, respectively. We found evidence of null alleles in two markers (MU05 and G10X) in the Eastern Cantabrian subpopulation (S3 Table). Homozygote excess was only found for one allele in each of these two markers, i.e., allele 129 in MU05 and allele 128 in G10X. After careful manual inspection of the electrophoretograms from replicate runs for MU05 and G10X, we confidently excluded the possibility of allele dropout, null alleles or stuttering for the Eastern Cantabrian subpopulation. Given that there is ongoing gene flow between the two subpopulations, recent migration patterns may have affected the proportions of homozygotes and heterozygotes in both subpopulations. Notably, the Eastern Cantabrian subpopulation is smaller, so it is more prone to exhibit such variation. There was no evidence of null alleles, stuttering or dropout for the Western Cantabrian subpopulation when first generation migrants were excluded from the analysis.

### Phylogeographic affinities

**Fig 2:**
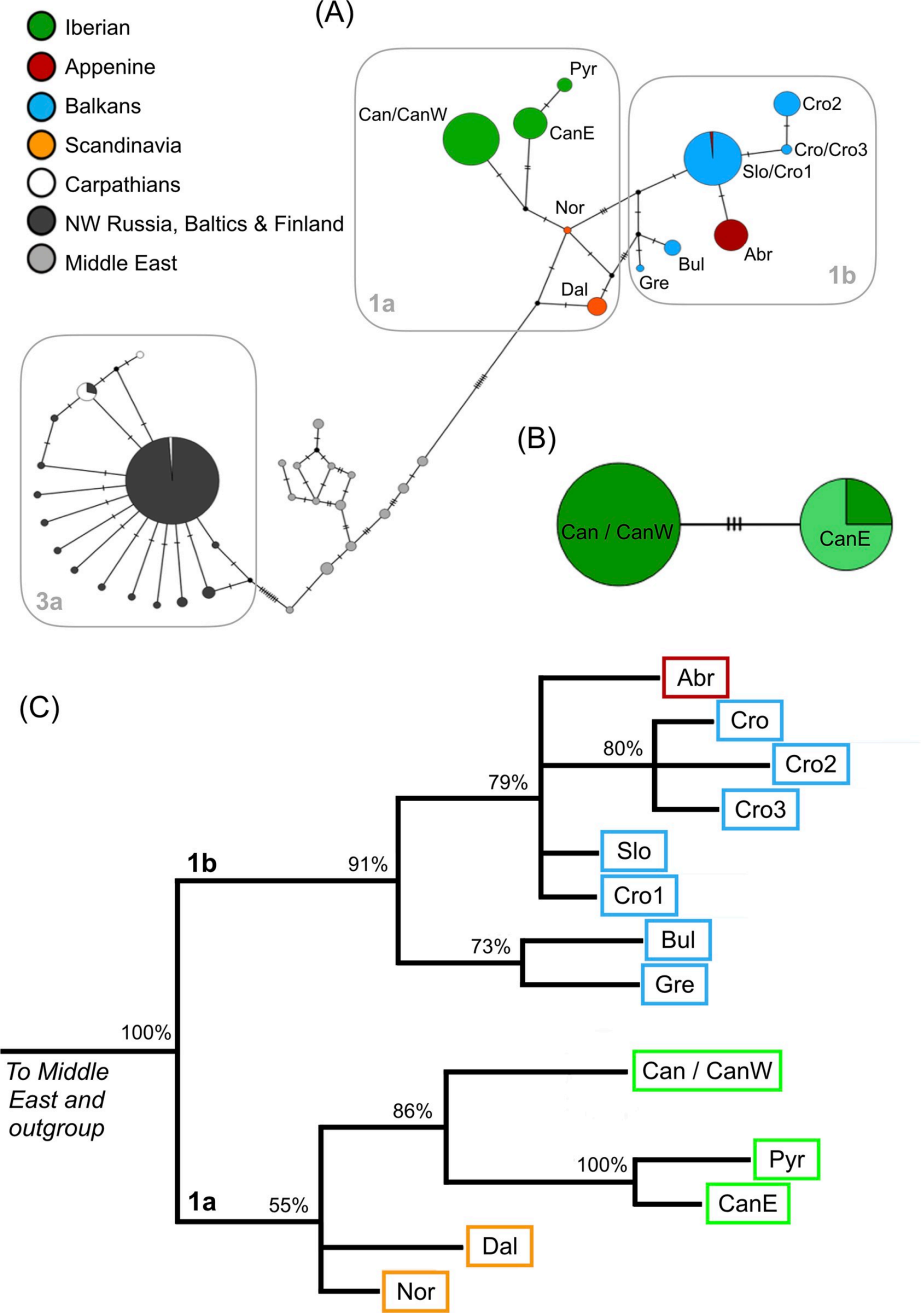
Phylogenetic and phylogeographic affinities of the Cantabrian brown bear with other European brown bear populations. (a) Median-joining network of 83 brown bear mtDNA haplotypes from across Europe and the Middle East. Haplotypes are colour-coded according to geographic origin, in agreement with the nomenclature given by Taberlet and Bouvet [14]. Iberian haplotypes have been named “CanW” and “CanE” according to their respective regions of origin in Cantabria. Mutational steps between haplotypes are represented by dashes. (b) Median-joining network of the two mtDNA haplotypes sampled from the Cantabrian population. Dark green corresponds to individuals sampled from the Western Cantabrian subpopulation and light green corresponds to individuals sampled from the Eastern Cantabrian subpopulation. (c) Details of the Bayesian inference tree based on 83 brown bear haplotypes from across Europe and the Middle East. Clade names follow Davison et al. [54].

In this study, we generated 112 new sequences for the mtDNA control region (including the 80 individual genotypes, recaptures and samples with insufficient microsatellite data for reconstructing genotypes), assigned to two different haplotypes, i.e. CanW and CanE (Fig 2B, Genbank accession numbers: MN477248 and MN477249). Haplotype CanW was only found in bears sampled from the Western Cantabrian subpopulation (n = 43). Haplotype CanE was recovered from all bears sampled in the Eastern Cantabrian subpopulation (n = 30), as well as in seven males (8OC, 14OC, 49OC, 71OC, 77OC, 92OC and 93OC, S4 Table) sampled from the Western Cantabrian subpopulation. The median-joining network (Fig 2C) revealed that haplotype CanW corresponds to haplotype “Can” previously reported by Taberlet and Bouvet [14]. Haplotype CanE is recorded for the first time in this study and is more closely related (a distance of one mutational step) to haplotype “Pyr” from the Pyrenees than it is to haplotype CanW (a distance of three mutational steps). The two Iberian haplotypes also appear to be more closely related to those from Scandinavia (Clade 1a) than to haplotypes from other peninsulas of southern Europe (Clade 1b), as reported in previous studies [14,54], though that relationship is not strongly supported by Bayesian inference. However, the relationship between CanE and Pyr haplotypes is strongly supported by Bayesian inference (posterior probability = 100%, Fig 2A; complete phylogeny in S1 Fig), suggesting that the Cantabrian population is not monophyletic.

### Genetic structure, connectivity and gene flow

Five of the 15 microsatellite loci we assessed significantly departed from HWE conditions, and 48 out of 105 pairwise combinations presented deviations to LE conditions (Table 1), for the whole Cantabrian population and after Bonferroni correction. Deviations from HWE and LE conditions were substantially reduced when each subpopulation was analysed separately; only two or three loci showed departures from HWE in both subpopulations, and the Western and Eastern Cantabrian subpopulations exhibited 4 or 27 pairs of loci with significant deviations from LE after Bonferroni correction, respectively (Table 1).

**Table 1:** Genetic diversity indices for the Cantabrian brown bear population and its subpopulations, based on 15 microsatellite markers. Numbers of loci or pairs of loci with significant deviations from Hardy-Weinberg equilibrium and linkage equilibrium conditions after Bonferroni correction are shown. Significant values are in italics.

		Population or sub-population			
	Parameter	*Cantabrian Mountains n = 80*	*Western n = 43*	*Western with Migrants n = 50*	*Eastern n = 30*
Structure	*Loci* in HWD	5/15	3/15	3/15	2/15
	LD (pairs of *loci* in LD)	48/105	4/105	15/105	27/105
Genetic Diversity	A	53	49	50	43
	A_p_	-	9	10	3
	A_r_	6.37	5.61	5.97	5.15
	Gene Diversity	0.525	0.544	0.490	0.516
	H_E_	0.539	0.487	0.513	0.508
	H_O_	0.481	0.509	0.515	0.453
Endogamy	F_IS_	0.073	-0.002	-0.017	*0*.*141*
Effective Population Sizes	N_e_ (95% CI)	-	24.7 (15.4–43.7)	10.1 (3.8–20.6)	1.8 (1.2–2.7)
	N_eb_ (95% CI)	3.5 (2.5–4.7)	4.1 (2.6–6.0)	5.3 (2.8–8.5)	2.1 (1.1–3.4)
Bottlenecks	M ratio	*0*.*614*	*0*.*620*	*0*.*627*	*0*.*610*
	Heterozygosity Excess* (p values)	*0.011/**0.001*	0.252/0.095	0.119/*0.022*	*0.003*/*0.003*

Abbreviations: HWD, deviations to Hardy-Weinberg equilibrium conditions; LD, linkage disequilibrium; A, number of alleles; A_p_, private alleles; A_r_, allele richness (rarefied); H_E_, expected heterozygosity; H_O_, observed heterozygosity; F_IS_, inbreeding coefficient; N_e_, effective population size; N_eb_, effective number of breeders.

* significance of excess: p values for Sign/Wilcoxon tests under a two phase model (TPM).

The genetic distance between the Western and Eastern Cantabrian subpopulations was always significant (p<0.001), ranging from F_ST_ = 0.145 when first generation migrants were included in the Western Cantabrian subpopulation to F_ST_ = 0.207 when migrants were excluded from the analysis. These values indicate considerable genetic differentiation between the two subpopulations [42], with structuring of the combined Cantabrian Mountains population being significant (p<0.001) whether or not migrants were included in the analysis. When we performed an AMOVA that included first generation migrants in the Western subpopulation, 82.4% of the total genetic differentiation was attributed to differences within individuals, 14.5% was attributed to differences among populations, and 3.1% to differences among individuals within subpopulations. When first generation migrants were removed from that analysis, 79.9% of the total genetic differentiation was attributed to differences within individuals, 18.6% was attributed to differences among populations, and 1.4% to differences among individuals within subpopulations.

Genotypes from the Cantabrian Mountains were consistently divided into two (K = 2) distinct genetic clusters with high posterior probabilities (data not shown). The results of the 10 replicate runs were highly consistent, including for *Q* proportions of the individual genotypes assigned to each of the inferred genetic clusters. There was strong agreement among the inferred genetic clusters and the geographic origin of sampled individuals (Western and Eastern Cantabrian subpopulations). Therefore, the two genetic clusters were denominated CanWest and CanEast, corresponding to sampling areas and known subpopulations. Individual genotypes were mostly assigned to the genetic cluster corresponding to the subpopulation from which the individuals were sampled.

**Fig 3:**
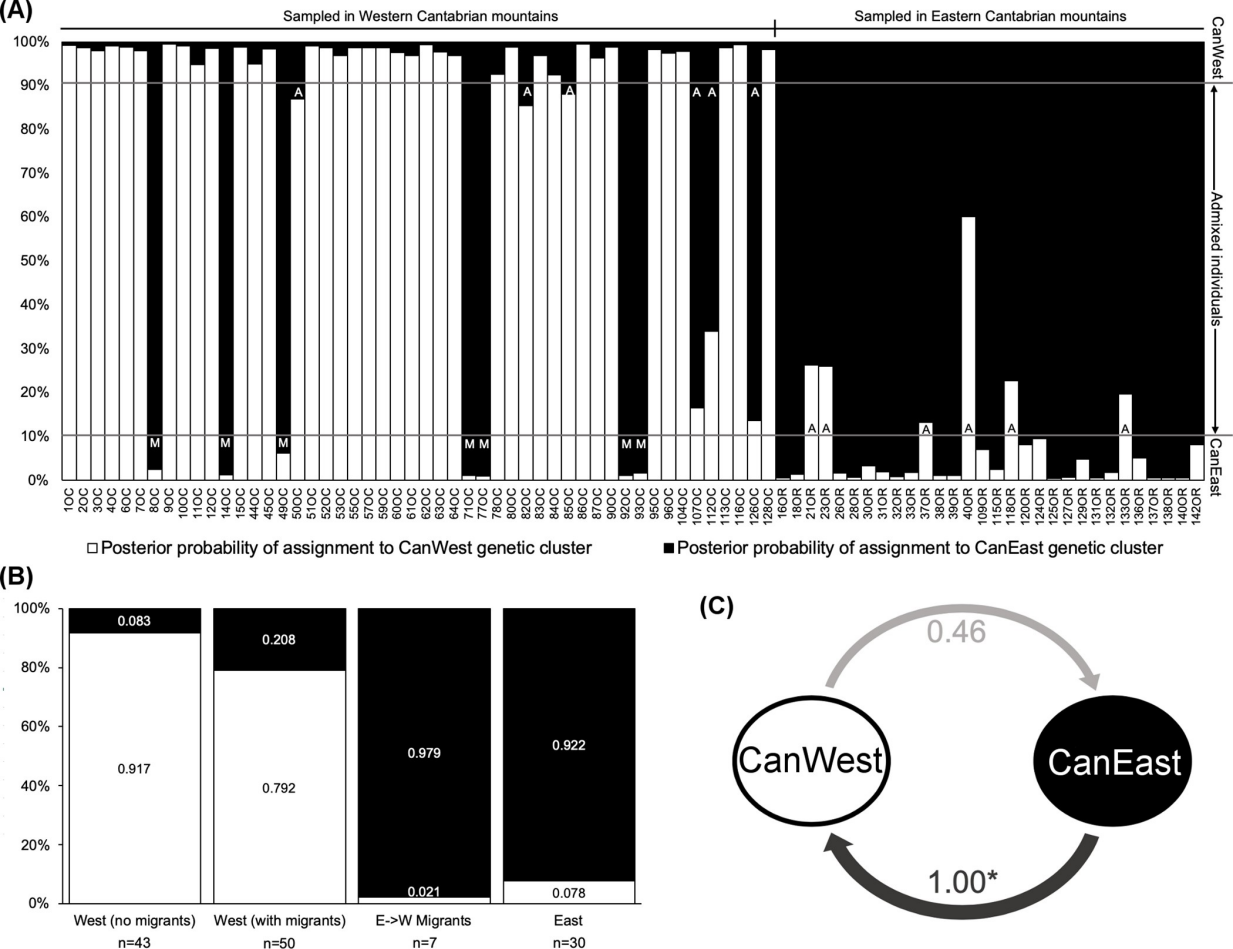
Genetic assignment, admixture and gene flow among brown bear subpopulations in the Cantabrian Mountains. (a) Posterior probability of assignment of individual genotypes to each of the genetic clusters matching the two subpopulations. Thresholds of assignment to the CanWest and CanEast genetic clusters are represented as horizontal lines. Admixed individuals (denoted “A”) have intermediate assignment posterior probabilities. First generation migrants (denoted “M”) were assigned with higher posterior probability to the subpopulation other than that from which they were sampled. (b) Average posterior probabilities of assignment to each genetic cluster per subpopulation, and for the Western Cantabrian subpopulation without migrants, or for first generation migrants alone. (c) Relative migration flows (in number of migrants, Nm). Significantly asymmetric migration flow is marked with an asterisk.

Estimated threshold values (HYBRIDLABS) for assigning individuals to either one of the two genetic clusters, corresponding to subpopulations, was Tq>0.90. Hence, we assigned all individuals with Tq>0.90 to one of the two subpopulations (Fig 3). Seven individuals (8OC, 14OC, 49OC, 71OC, 77OC, 92OC and 93OC) sampled from the Western Cantabrian subpopulation were assigned with significant posterior probability to the CanEast genetic cluster (Fig 3). Coincidentally, these individuals correspond to seven males with the CanE mitochondrial haplotype that had been captured in the Western Cantabrian region. Based on their mtDNA lineages and posterior probabilities of genotype assignment, these individuals were considered as first generation migrants (denoted “M” in Fig 3). Twelve additional individuals (denoted “A” in Fig 3) were not assigned with significant posterior probabilities to either of the two subpopulations (Fig 3A). Therefore, all 12 of those individuals were considered to be of admixed origin. However, in all 12 cases, the haplotype of the individuals matched the subpopulation from which they were sampled.

**Fig 4:**
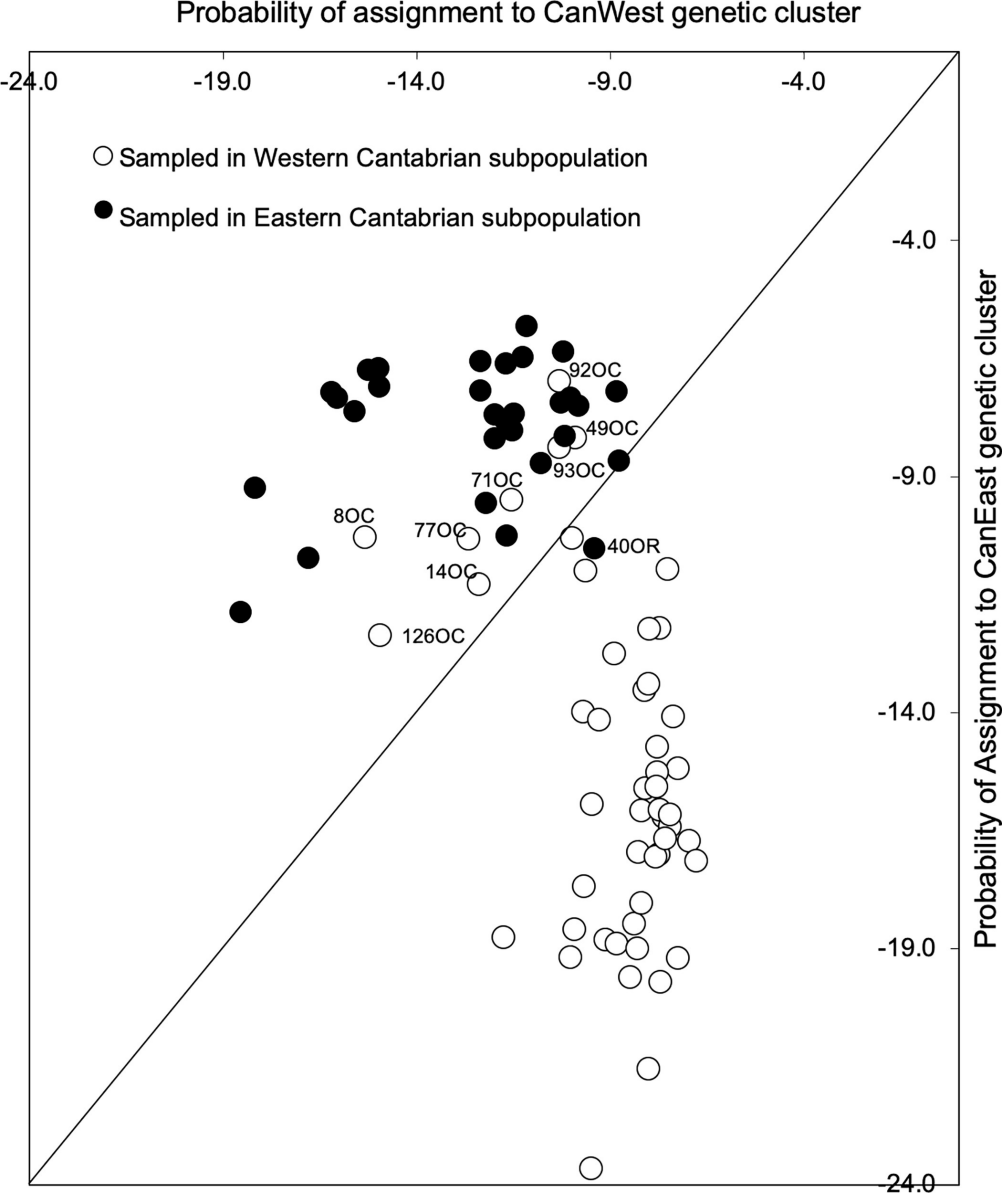
Probability of assigning each individual sampled from the Western and Eastern Cantabrian subpopulations to the CanEast and CanWest genetic clusters. Less negative values correspond to higher assignment probabilities.

Overall, the results from assignments performed using GENALEX (Fig 4) were in agreement with the results from STRUCTURE, with all first generation migrants being assigned to the CanEast genetic cluster despite having been sampled in the territory of the Western subpopulation. In order to exclude the possibility of assignment probabilities being influenced by the unbalanced sampling sizes of the Western and Eastern Cantabrian subpopulations, we repeated the assignment tests for three randomly rarefied sample sets (n = 30) of the Western Cantabrian subpopulation and consistently obtained the same patterns of assignment. Moreover, the same set of seven first-generation migrants was consistently detected using BAYESASS and GENECLASS (S5 Table). Additional individuals were also identified with high probability as being first-generation migrants in BAYESASS (126OC) or in GENECLASS without applying a simulation algorithm (126OC, 23OR, 40OR). However, when we did apply a simulation algorithm in GENECLASS, only five individuals were identified as being first-generation migrants (8OC, 77OC, 92OC, 126OC, 40OR). Accordingly, as a conservative approach, we only considered individuals to be first-generation migrants when identified as such at least once in each approach and possessed a mtDNA haplotype inconsistent with their capture location.

Relative migration network patterns were the same, regardless of the differentiation statistic (Nm, D or Gst) implemented in the R package. Relative migration flows (in Nm–number of migrants) were detected in both directions between the Western and Eastern Cantabrian subpopulations. However, a significant asymmetric relative migration flow was detected from the Eastern to the Western subpopulation (Fig 3C). These results are consistent with the migration rates estimated using BAYESASS (S5 Table).

### Genetic diversity and demographic parameters

Rarefied allelic richness was 6.37 for the whole Cantabrian population (Table 1), but it was higher in the Western Cantabrian subpopulation with (5.97) or without migrants (5.61) than in the Eastern Cantabrian subpopulation (5.15). Expected heterozygosity (H_E_) was higher in the Western Cantabrian subpopulation including migrants (0.513) than in the Eastern Cantabrian subpopulation (0.508). Observed heterozygosity (H_O_) was higher than expected (0.515) in the Western Cantabrian subpopulation (including migrants), but lower than expected (0.453) in the Eastern Cantabrian subpopulation. The entire Cantabrian population exhibits a significant heterozygosity deficit (H_E_>H_O_), most likely related to the presence of genetic structure. The inbreeding coefficient was positive for the entire population in the Cantabrian Mountains, it was marginally negative for the Western Cantabrian subpopulation, and it was positive and significant for the Eastern Cantabrian subpopulation. The differences between the two subpopulations are consistent with the estimates of inbreeding coefficients obtained using BAYESASS (S5 Table).

We did not estimate N_e_ for the entire Cantabrian population because population structure results in biased N_e_ estimation using the Linkage Disequilibrium method. We estimated N_e_ of 1.8 for the Eastern Cantabrian subpopulation and 24.7 for the Western Cantabrian subpopulation (excluding migrants). N_eb_ was 2.1 for the Eastern Cantabrian subpopulation and 5.3 for the Western Cantabrian subpopulation (including migrants) (Table 1).

As expected for bottlenecked populations [44], we observed heterozygosity excess in the entire Cantabrian population, as well as in both subpopulations, based on both Wilcoxon and Sign tests (Table 1). Heterozygosity excess was significant (p<0.05) for the Wilcoxon test in all cases, except for the Western Cantabrian subpopulation without migrants, for which it was only marginally significant (p<0.1). Sign tests were significant for heterozygosity excess (p<0.05) only for the entire Cantabrian population and for the Eastern Cantabrian subpopulation. M ratio values for the entire population and all subpopulations were also significantly below average and critical ratio values expected for stable populations.

## Discussion

### Origins and phylogeographic affinities

Our results have helped clarify the phylogeographic relationships of the Cantabrian brown bear population in relation to other Iberian and European populations. Previous studies have reported the existence of two main mitochondrial DNA lineages in Eurasia [14,54], corresponding to Western Eurasian (Clade 1, comprising two subclades: 1a and 1b) and Eastern Eurasian lineages (Clade 3a). In those studies, the Cantabrian brown bear population was included in the Western Eurasian lineage (more specifically in Clade 1a), which is closely related to the Pyrenean population [13,14,15]. However, we found that the Cantabrian brown bear population is not monophyletic, haplotypes Can/CanW and CanE are not sister groups and are geographically structured. Nevertheless, both haplotypes belong to the Western Eurasian lineage (Clade 1a) [14,54]. Haplotype CanE is more closely related to the Pyr haplotype previously reported in Taberlet and Bouvet [14] than it is to Can/CanW, indicating that the Eastern Cantabrian subpopulation is more closely related to the historical brown bear population of the Pyrenees. The current Pyrenean population is primarily derived from individuals translocated from Slovenia in 1995 and, currently, there is no evidence that the original Pyrenean population persisted after the translocation event [55]. Thus, it is likely that the current Pyrenean brown bear population is genetically more similar to the Slovenian population [3,56], and the closest population to historical Pyrenean brown bears could actually be the Eastern Cantabrian subpopulation.

During the Last Glacial Maximum (LGM), several mammal species found refuge in southern European peninsulas [57]. For certain species, mtDNA phylogenetic patterns reveal differentiation within those peninsulas, with some populations being more closely related to central and north European populations than they are to other peninsular populations. This pattern has been observed for Iberian populations of roe deer, *Capreolus capreolus* [58,59] and wild boar, *Sus scrofa* [60,61]. Here, we found a similar east-west differentiation pattern for brown bears in northwestern Iberia. That phylogeographic pattern may be consistent with the movement of populations from northern European regions into the peninsulas during the LGM, driving original pre-LGM populations further into the peninsulas [60]. Where those populations persisted in the peninsulas, it is possible to observe phylogenetic lineages with different affinities.

Similarly, it is also conceivable that the differences we report for the Cantabrian brown bear population could result from identical population dynamics occurring before and during the LGM. According to this latter hypothesis, the Western Cantabrian subpopulation (represented by the haplotype CanW) would represent a remnant of the pre-LGM Cantabrian population. A different population coming from the east (possibly from the Pyrenees and currently represented by the haplotype CanE) could have driven this population westward during the LGM. Thus, contemporary Eastern Cantabrian bears may be descended from bears that secondarily colonized the Cantabrian Mountains via the Pyrenees. It is important to emphasize that despite being closer to the Pyr haplotype, the CanE haplotype differs from the latter by one mutational step, which is consistent with patterns observed for other species (see [60]). Valdosiera et al. [62] raised the possibility that Iberian brown bear populations received genetic contributions from other European populations, more specifically via mitochondrial gene flow, based on analyses of ancient brown bear DNA. Curiously, when we compared the haplotypes detected in this study with those identified by Valdosiera et al. [62], we found that: (a) haplotype CanW matched the Cantabrian haplotype first reported by Taberlet and Bouvet [14] that was related to other haplotypes exclusively sampled within Iberia; and (b) CanE still stood out as a previously unreported haplotype that was more closely related to haplotypes sampled in Iberia, the Pyrenees and France (S2 Fig).

These findings reinforce the urgency of preserving the Cantabrian population, which might represent the only remnant of more ancient and diverse Iberian lineages. The potentially distinct origins of the two Cantabrian subpopulations have not prevented historical gene flow between them, as discussed in more detail below. However, gene flow in brown bear populations is mostly mediated by male dispersal, so it has limited impact on patterns of matrilineal (mtDNA) lineages [63,64].

### Genetic structure, diversity and health

The patterns identified from Cantabrian brown bear mitochondrial lineages provide further support for dividing this population into two genetic clusters (CanWest and CanEast), as also established here (and in previous studies) using nuclear recombinant markers. These genetic clusters strongly match the Western and Eastern Cantabrian subpopulations, which are strongly differentiated from each other. According to previous authors [8,65,66,67], this differentiation might be due to strongly reduced connectivity between the subpopulations nearly two centuries ago. Our results suggest that apart from this more recent isolation, the two subpopulations might be descended from historically distinct populations. The genetic diversity of both Cantabrian brown bear subpopulations appears to have been increasing over recent years (Table 2) [62], even though their observed diversity remains low when compared with other European populations such as the Scandinavian brown bear population (H_o_ = 0.82; [68]). There is also evidence that diversity of the Iberian brown bear population has decreased from the Pleistocene to modern times [62].

**Table 2:** Summary of the genetic diversity of the two Cantabrian brown bear subpopulations obtained from different studies.

	*Period of study (years)*	*No*. *of genotypes (loci) used*	*H*_*o*_	*F*_*IS*_	*Reference*
Western subpopulation	2002–2003	91 (≥11)	0.49	-	García-Garitagoitia et al. 2006 (in [67])
	2006–2008	31 (≥14)	0.44	-	[8]
	2013–2014	12 (≥16)	0.49	0.026	[67]
	2010–2017	50 (≥12*)	0.515	-0.017	This study
Eastern Subpopulation	1996–1997	20 (≥8)	0.36	-	Rey et al. 2000 (in [67])
	1991–1999	27 (≥11)	0.47	-	García-Garitagoitia et al. 2006 (in [67])
	2006–2008	9 (≥14	0.28	-	[8]
	2013–2014	26 (≥16)	0.54	0.038	[67]
	2010–2017	30 (≥12*)	0.453	0.141	This study

* Despite our analytical threshold of 12 loci, most genotypes were reconstructed based on ≥14 loci (average 14.5).

The strong evidence we found of bottlenecks in the Cantabrian subpopulations may explain their low genetic diversity. Higher genetic diversity is normally associated with more stable populations, having larger population sizes, such as those observed in Scandinavia [16,17]. Therefore, the low genetic diversity observed in the Cantabrian population can be linked to its isolation from other European brown bear populations and its fragmented nature [6]. Low genetic diversity, particularly in small and isolated populations, is often related with higher extinction risk. Nevertheless, a recent study revealed that the small and isolated Apennine brown bear population was able to survive for several millennia, accumulating deleterious mutations, mostly by drift, while preserving variation in key regions of the genome associated with immune system and olfactory perception [69]. Drift is stronger in smaller populations and, thus, the small population sizes of the Cantabrian subpopulation can also contribute to lower genetic diversity. Recent studies have estimate population sizes of ~200 individuals for the Western Cantabrian subpopulation and ~19–30 individuals for the Eastern Cantabrian subpopulation [11,70]. We identified a minimum number of 43 individuals in the Western Cantabrian subpopulation (50 individuals if migrants are also considered), and a minimum number of 30 individuals in the Eastern Cantabrian subpopulation. It is important to note that these numbers must not be interpreted as census sizes for these two subpopulations since the sampling time-span and sample size are not adequate for such estimates, in particular for the Western Cantabrian population. Among other causes of population decline, it is possible that the Eastern Cantabrian subpopulation is actually “losing” individuals to the Western Cantabrian subpopulation. Our estimates also show a large difference in the effective population sizes of the Western (N_e_ = 24.7) and Eastern (N_e_ = 1.8) Cantabrian subpopulations. However, these results should be interpreted with caution since there are several methods for estimating effective population sizes, which are based on different time-scales and initial assumptions [71].

### Gene flow and dispersal of individuals

Our results reveal evidence of migration between the two Cantabrian subpopulations. There is solid proof of recent bear migration from the Eastern to Western subpopulation, since seven male individuals sampled in the West were assigned with high posterior probability to the Eastern Cantabrian subpopulation and possessed the CanE haplotype. Moreover, our results show that gene flow operates in both directions, but with a higher level of allelic introgression from the Eastern Cantabrian subpopulation into the Western Cantabrian subpopulation, a finding corroborated both by mitochondrial and genotype data. In fact, migration (gene flow) estimates based on allele frequencies (available in S6 Table) and on genotypes (S5 Table) are indicative of long-term and on-going asymmetrical gene flow from the Eastern to the Western Cantabrian subpopulation, contradicting previous studies that reported stronger gene flow in the opposite direction [8,67]. Together with our identification of first generation migrants from the Eastern into the Western Cantabrian Mountains, these results suggest that current migration is likely to be more intense from East to West.

Long-term monitoring of these populations [70] shows that the Western Cantabrian subpopulation (n = 200) is considerably larger than the Eastern Cantabrian subpopulation (n = 25 to 30), suggesting that the previously reported west-to-east gene flow across the Cantabrian Mountains could support recovery of the Eastern Cantabrian subpopulation through the arrival of reproductive individuals from the Western Cantabrian subpopulation. However, our results provide strong evidence for male migration from the Eastern Cantabrian subpopulation into the Western Cantabrian subpopulation, i.e. against the gradient of population size. A similar pattern of asymmetrical male-mediated migration was recently reported for brown bears in the Carpathian mountains [72].

From an ecological point of view (based on source-sink dynamics), this east-to-west bias may seem contradictory, as it could be reasonably assumed that the more stable and larger population (Western Cantabrian) would act as a source, and disperse into the smaller, less stable and more fragmented sink population (Eastern Cantabrian) [73]. Nevertheless, we assert that our findings are not counter-intuitive, and present three non-mutually exclusive hypotheses that could explain the migration of brown bears from the Eastern into the Western Cantabrian subpopulation.

First, the migration pattern could be explained by there being an unfavourable sex ratio in the Eastern Cantabrian subpopulation, leading to male dispersal into Western territories where the number of females is higher. Although we did not find significant differences in sex ratio in this study, long-term monitoring of the brown bear subpopulations in the Cantabrian Mountains [71] suggests that the Western subpopulation harbours eight to ten times the number of reproductive females (n~34) than the Eastern subpopulation (n~4). Second, more favourable habitats in the Western Cantabrian Mountains could explain asymmetric gene flow. However, based on our intimate knowledge of the study area and the fact that brown bears are currently absent from Eastern Cantabrian territories deemed highly suited to them, we feel there is less support for this hypothesis. Third, brown bears could be forced to flee from areas with higher human disturbance and poaching, which are more intense in the Eastern Cantabrian Mountains. In fact, a recent study by Lamamy and collaborators [74] has revealed that differences on population trend, numbers and fecundity among both subpopulations cannot be explained by differences in habitat or landscape alone and might result from direct human influence (e.g. poaching and bad hunting practices). Hence, it is reasonable to infer that individuals from the Eastern Cantabrian subpopulation might be dispersing westwards to seek habitats with less human interference and to escape human persecution. Ecological modelling of brown bear presence and distribution in relation to human activities and other critical factors might shed light on the main drivers of brown bear dispersal and gene flow within the Cantabrian Mountains.

### Implications for conservation

The above-presented hypotheses may explain in part the apparently unexpected dispersal of brown bears from east-to-west across the Cantabrian Mountains. While reinforcing previous findings that brown bears do move between Cantabrian subpopulations, our findings suggest they might be dispersing from the smaller Eastern subpopulation towards the larger and more stable Western subpopulation. Thus, instead of promoting colonization (and reinforcement) of the Eastern Cantabrian region by bears from the Western Cantabrian Mountains, connectivity between the two subpopulations may operate as a means for Eastern Cantabrian bears to find more suitable territories.

Undoubtedly in this specific case, suitable corridors across the Cantabrian landscape are vital to bolstering the connectivity between both subpopulations. Whether increased connectivity is a consequence of active habitat improvement measures or shifting land use patterns (e.g. progressive abandonment of historic mining activity), it is unlikely that enhancing subpopulation connectivity alone will serve to restore these two threatened subpopulations. Other conservation measures should be implemented in order to promote settlement of individuals in the Eastern Cantabrian Mountains. The ecological requirements of brown bears are well known, so appropriate habitat restoration could be implemented that would increase the carrying capacity of Eastern Cantabrian territories [73]. We feel that more efficient control over poaching and other direct human persecution, as well as greater efforts to raise public awareness in order to reduce human-bear conflict, might be even more successful as a means to secure the Eastern Cantabrian subpopulation. Simultaneous implementation of both these measures could improve the effective size, diversity and status of the brown bear subpopulations across the Cantabrian Mountains, thereby ensuring their future viability. Our results shed light on the historical affinities of the Cantabrian subpopulations of brown bears in an Iberian context, and provide new insights into the genetic health and migration patterns of the Cantabrian brown bear population. Our data can help with evaluations of conservation strategies implemented for the brown bear population in the Cantabrian Mountains and in defining new strategies to maintain a viable population in that region. It also provides useful information relevant to monitoring expansions of brown bear populations, especially given recent report of brown bear sightings and damage to apiaries in North Portugal, by national conservancy agency (ICNF) rangers.

## Supporting information

S1 Fig Complete Bayesian inference tree. Bayesian support for nodes (posterior probability) is presented in front of each node.
(PDF)

S2 Fig Median-joining networks reconstructed using brown bear modern and ancient (Holocene and Pleistocene) samples. (PDF)

S1 Table Multilocus genotypes of 81 brown bears from the Cantabrian Mountains. (XLSX)

S2 Table Details of the mitochondrial DNA sequences (retrieved from GenBank) used in our phylogeographic and phylogenetic analyses. (PDF)

S3 Table Evidence for null alleles, allele drop-out and stuttering from microsatellite marker genotyping, retrieved from MICROCHECKER analyses. (PDF)

S4 Table Sampling location, haplotype and sex of the individuals sampled in the Cantabrian Mountains. (PDF)

S5 Table Estimation of migration rates and migrant identification using BAYESASS and GENECLASS. (PDF)

S6 Table Allele frequencies per loci, for the entire Cantabrian population, the Eastern and Western subpopulations. (PDF)
