# Supplementary material for: Paths for colonization or exodus? New insights from the brown bear (Ursus arctos) population of the Cantabrian Mountains
Source: PLoS One. 2020 Jan 31;15(1):e0227302. doi: 10.1371/journal.pone.0227302 (PMC6996475; doi:10.1371/journal.pone.0227302)
Supplement: S1 Fig — Bayesian support for nodes (posterior probability) is presented in front of each node. (PDF) [file pone.0227302.s001.pdf]

SUPPORTING INFORMATION S5 - Complete Bayesian inference tree. Bayesian support for nodes (posterior probability) is presented in front of each node.

Gregório, I, Barros, T, Pando, D, Morante, J, Fonseca, C, Ferreira, E (2019). A path for colonization or exodus? New insights from the Cantabrian brown bear population. PLOS One (submitted).

Eduardo Ferreira (Corresponding author, e-mail: elferreira@ua.pt). Department of Biology & CESAM, University of Aveiro, Campus Universitário de Santiago, 3810-193 Aveiro, Portugal.

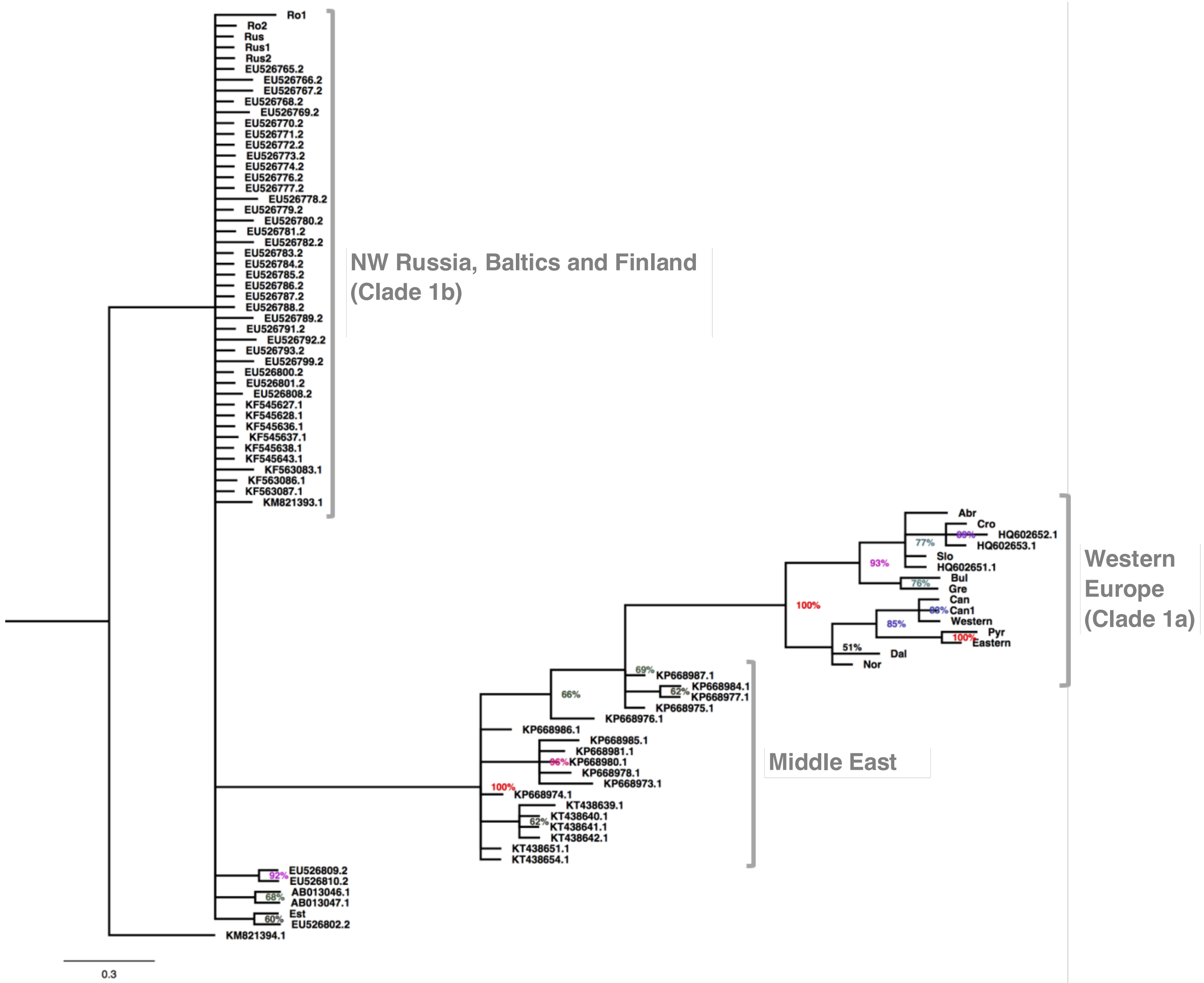

Figure S5. Complete Bayesian inference tree constructed with all haplotypes used in this study. Bayesian support for nodes (posterior probability) is presented in front of each node. Clade names follow Davison et al [54].
