# Supplementary material for: Paths for colonization or exodus? New insights from the brown bear (Ursus arctos) population of the Cantabrian Mountains
Source: PLoS One. 2020 Jan 31;15(1):e0227302. doi: 10.1371/journal.pone.0227302 (PMC6996475; doi:10.1371/journal.pone.0227302)

SUPPORTING INFORMATION S7 - Median-joining networks reconstructed using brown bear modern and ancient (Holocene and Pleistocene) samples.

Gregório, I, Barros, T, Pando, D, Morante, J, Fonseca, C, Ferreira, E (2019). A path for colonization or exodus? New insights from the Cantabrian brown bear population. PLOS One (submitted).

Eduardo Ferreira (Corresponding author, e-mail: elferreira@ua.pt). Department of Biology & CESAM, University of Aveiro, Campus Universitário de Santiago, 3810-193 Aveiro, Portugal.

Figure S7 - Median-joining networks reconstructed using the brown bear haplotypes (and haplotype frequencies) found in this study (CanW and CanE), as well as modern and ancient (Holocene and Pleistocene) brown bear haplotypes reported from Iberia in Valdosiera et al. [62]. Networks were generated using the long (Figure S7.A) and short (Figure S7.B) datasets used by Valdosiera and collaborators in their study. Haplotype names (Ua4 to Ua29) follow the naming convention applied in Figure 1B of Valdosiera et al. [62] (and haplotypes 1-25 in brackets in Figure 1A). Haplotype names used in our study (CanW and CanE) and in Taberlet & Bouvet [14] are also shown for reference.

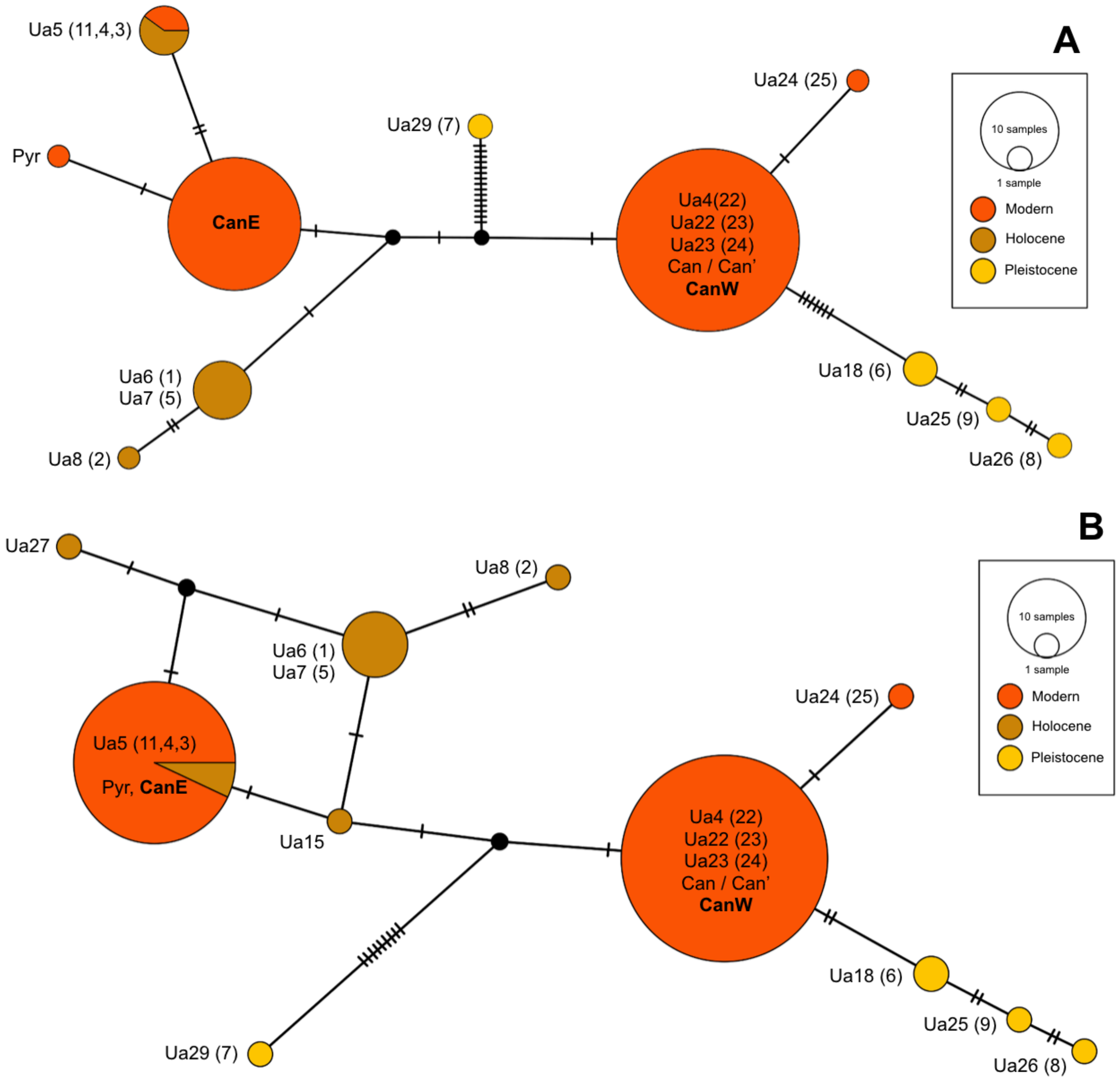

Supplement: S2 Fig — (PDF) [file pone.0227302.s002.pdf]
