# Supplementary material for: Paths for colonization or exodus? New insights from the brown bear (Ursus arctos) population of the Cantabrian Mountains
Source: PLoS One. 2020 Jan 31;15(1):e0227302. doi: 10.1371/journal.pone.0227302 (PMC6996475; doi:10.1371/journal.pone.0227302)
Supplement: S2 Table — (PDF) [file pone.0227302.s004.pdf]

SUPPORTING INFORMATION S2 - Details of the mitochondrial DNA sequences (retrieved from GenBank) used in our phylogeographic and phylogenetic analyses.

Gregório, I, Barros, T, Pando, D, Morante, J, Fonseca, C, Ferreira, E (2019). A path for colonization or exodus? New insights from the Cantabrian brown bear population. *PLOS One* (submitted)

Eduardo Ferreira (Corresponding author, e-mail: elferreira@ua.pt). Department of Biology & CESAM, University of Aveiro, Campus Universitário de Santiago, 3810-193 Aveiro, Portugal

Table S2 - Details of the mitochondrial DNA sequences (retrieved from GenBank) used in our phylogeographic and phylogenetic analyses.

GenBank Acession No.	Location	Reference
X75862.1	Abruzzo, Italy	Taberlet & Bouvet 1994
X75864.1	Bulgary	Taberlet & Bouvet 1994
X75865.1	Cantabria, Spain	Taberlet & Bouvet 1994
X75866.1	Cantabria, Spain	Taberlet & Bouvet 1994
X75867.1	Croatia	Taberlet & Bouvet 1994
X75868.1	Sweden	Taberlet & Bouvet 1994
X75869.1	Estonia	Taberlet & Bouvet 1994
X75870.1	Greece	Taberlet & Bouvet 1994
X75871.1	Norway	Taberlet & Bouvet 1994
X75872.1	Romania	Taberlet & Bouvet 1994
X75873.1	Romania	Taberlet & Bouvet 1994
X75874.1	Estonia, Sweden, Finland, Russia	Taberlet & Bouvet 1994
X75875.1	Slovakia	Taberlet & Bouvet 1994
X75876.1	Slovakia	Taberlet & Bouvet 1994
X75877.1	Trentino, Italy	Taberlet & Bouvet 1994
X75878.1	Pyrenees, France	Taberlet & Bouvet 1994
EU526765.2	Estonia, Finland, European Russia	Korsten et al. 2009
EU526766.2	European Russia	Korsten et al. 2009
EU526767.2	Finland	Korsten et al. 2009
EU526768.2	European Russia	Korsten et al. 2009
EU526769.2	European Russia	Korsten et al. 2009
EU526770.2	Finland, European Russia	Korsten et al. 2009
EU526771.2	European Russia	Korsten et al. 2009
EU526772.2	European Russia	Korsten et al. 2009
EU526773.2	Finland	Korsten et al. 2009
EU526774.2	European Russia	Korsten et al. 2009
EU526776.2	European Russia	Korsten et al. 2009
EU526777.2	Finland, European Russia	Korsten et al. 2009
EU526778.2	Finland	Korsten et al. 2009
EU526779.2	Finland	Korsten et al. 2009
EU526780.2	Finland	Korsten et al. 2009
EU526781.2	European Russia	Korsten et al. 2009
EU526782.2	European Russia	Korsten et al. 2009
EU526783.2	European Russia	Korsten et al. 2009
EU526784.2	Estonia	Korsten et al. 2009

EU526785.2	Estonia, European Russia	Korsten et al. 2009
EU526786.2	European Russia	Korsten et al. 2009
EU526787.2	European Russia	Korsten et al. 2009
EU526788.2	European Russia	Korsten et al. 2009
EU526789.2	European Russia	Korsten et al. 2009
EU526791.2	European Russia	Korsten et al. 2009
EU526792.2	Finland	Korsten et al. 2009
EU526793.2	Finland, European Russia	Korsten et al. 2009
EU526799.2	Finland	Korsten et al. 2009
EU526800.2	Russia	Korsten et al. 2009
EU526801.2	Estonia	Korsten et al. 2009
EU526802.2	Estonia	Korsten et al. 2009
EU526808.2	Russia	Korsten et al. 2009
EU526809.2	Russia	Korsten et al. 2009
EU526810.2	Russia	Korsten et al. 2009
HQ602651.1	Croatia	Kocijan et al. 2011
HQ602652.1	Croatia	Kocijan et al. 2011
HQ602653.1	Croatia	Kocijan et al. 2011
KF545627.1	Russia	Salomishkina et al. 2014
KF545628.1	Russia	Salomishkina et al. 2014
KF545636.1	Russia	Salomishkina et al. 2014
KF545637.1	Russia	Salomishkina et al. 2014
KF545638.1	Russia	Salomishkina et al. 2014
KF545643.1	Russia	Salomishkina et al. 2014
KF563083.1	Russia	Salomishkina et al. 2014
KF563086.1	Russia	Salomishkina et al. 2014
KF563087.1	Russia	Salomishkina et al. 2014
KP668987.1	Iran	Ashrafzadeh et al. 2016
KP668986.1	Iran	Ashrafzadeh et al. 2016
KP668985.1	Iran	Ashrafzadeh et al. 2016
KP668984.1	Iran	Ashrafzadeh et al. 2016
KP668981.1	Iran	Ashrafzadeh et al. 2016
KP668980.1	Iran	Ashrafzadeh et al. 2016
KP668978.1	Iran	Ashrafzadeh et al. 2016
KP668977.1	Iran	Ashrafzadeh et al. 2016
KP668976.1	Iran	Ashrafzadeh et al. 2016
KP668975.1	Iran	Ashrafzadeh et al. 2016
KP668974.1	Iran	Ashrafzadeh et al. 2016
KP668973.1	Iran	Ashrafzadeh et al. 2016
KT438639.1	Turkey	Cilingir et al. 2016
KT438640.1	Turkey	Cilingir et al. 2016
KT438641.1	Turkey	Cilingir et al. 2016
KT438642.1	Turkey	Cilingir et al. 2016
KT438651.1	Turkey	Cilingir et al. 2016
KT438654.1	Turkey	Cilingir et al. 2016
AB013046.1*	Japan	Matsuhashi et al. 1999
AB013047.1*	Japan	Matsuhashi et al. 1999
KM821394.1*	Alaska	Talbot et al. (unpublished)

*-Sequences used as outgroup for Bayesian inference
