# Supplementary material for: Paths for colonization or exodus? New insights from the brown bear (Ursus arctos) population of the Cantabrian Mountains
Source: PLoS One. 2020 Jan 31;15(1):e0227302. doi: 10.1371/journal.pone.0227302 (PMC6996475; doi:10.1371/journal.pone.0227302)
Supplement: S3 Table — (PDF) [file pone.0227302.s005.pdf]

SUPPORTING INFORMATION S3 - Evidence for null alleles, allele drop-out and stuttering from microsatellite marker genotyping, retrieved from MICROCHECKER analyses.

Gregório, I, Barros, T, Pando, D, Morante, J, Fonseca, C, Ferreira, E (2019). A path for colonization or exodus? New insights from the Cantabrian brown bear population. PLOS One (submitted).

Eduardo Ferreira (Corresponding author, e-mail: elferreira@ua.pt). Department of Biology & CESAM, University of Aveiro, Campus Universitário de Santiago, 3810-193 Aveiro, Portugal.

Table S3. Evidence for null alleles, allele drop-out and stuttering from microsatellite marker genotyping, retrieved from MICROCHECKER analyses. (- no presence of allele dropout, null alleles nor stuttering).

Marker	Cantabrian Eastern	Cantabrian Western with migrants	Cantabrian Western without migrants
MU50	-	-	-
MU09	-	-	-
MU10	-	-	-
MU23	-	-	-
G10C	-	-	-
MU59	-	-	-
MU05	Null alleles	-	-
G10L	-	-	-
G1D	-	-	-
G10P	-	Null alleles	-
G10X	Null alleles	Null alleles and stuttering	-
G10J	-	-	-
MU51	-	-	-
G1A	-	-	-
MU61	-	-	-
