# Supplementary material for: Paths for colonization or exodus? New insights from the brown bear (Ursus arctos) population of the Cantabrian Mountains
Source: PLoS One. 2020 Jan 31;15(1):e0227302. doi: 10.1371/journal.pone.0227302 (PMC6996475; doi:10.1371/journal.pone.0227302)
Supplement: S4 Table — (PDF) [file pone.0227302.s006.pdf]

SUPPORTING INFORMATION S4 - Sampling location, haplotype and sex of the individuals sampled in the Cantabrian Mountains.

Gregório, I, Barros, T, Pando, D, Morante, J, Fonseca, C, Ferreira, E (2019). A path for colonization or exodus? New insights from the Cantabrian brown bear population. PLOS One (submitted).

Eduardo Ferreira (Corresponding author, e-mail: elferreira@ua.pt). Department of Biology & CESAM, University of Aveiro, Campus Universitário de Santiago, 3810-193 Aveiro, Portugal.

Table S4. Sampling location, haplotype and sex (as determined by molecular sex determination based on Amelogenin and SRY markers) of the individuals sampled form the Western and Eastern Cantabrian subpopulations.

Individual	Population	Sex	mtDNA Haplotype	Municipality (Recapture)	Province
1OC	Cantabrian Western	XX	W	Belmonte de Miranda	Asturias
2OC	Cantabrian Western	XY	W	Belmonte de Miranda	Asturias
3OC	Cantabrian Western	XY	W	Proaza	Asturias
4OC	Cantabrian Western	XX	W	Teverga	Asturias
6OC	Cantabrian Western	NA	W	Somiedo	Asturias
7OC	Cantabrian Western	XY	W	Proaza (Proaza)	Asturias
8OC	Cantabrian Western	XY	E	Villablino	Leon
9OC	Cantabrian Western	XY	W	Cangas de Narcea	Asturias
10OC	Cantabrian Western	NA	W	Proaza	Asturias
11OC	Cantabrian Western	NA	W	Proaza	Asturias
12OC	Cantabrian Western	XY	W	Teverga	Asturias
14OC	Cantabrian Western	XY	E	Somiedo (Belmonte de Miranda)	Asturias
15OC	Cantabrian Western	XX	W	Belmonte de Miranda	Asturias
44OC	Cantabrian Western	XY	W	Proaza	Asturias
45OC	Cantabrian Western	XY	W	Teverga	Asturias
49OC	Cantabrian Western	XY	E	Aller	Asturias
50OC	Cantabrian Western	XY	W	Teverga	Asturias
51OC	Cantabrian Western	NA	W	Somiedo	Asturias
52OC	Cantabrian Western	XX	W	Somiedo	Asturias
53OC	Cantabrian Western	XY	W	Somiedo (Belmonte de Miranda)	Asturias
55OC	Cantabrian Western	XY	W	Somiedo	Asturias
57OC	Cantabrian Western	XY	W	Somiedo (Somiedo)	Asturias
59OC	Cantabrian Western	XY	W	Somiedo	Asturias
60OC	Cantabrian Western	XY	W	Villablino	Leon
61OC	Cantabrian Western	XY	W	Villablino	Leon
62OC	Cantabrian Western	XY	W	Belmonte de Miranda	Asturias
63OC	Cantabrian Western	XX	W	Somiedo (Somiedo)	Asturias
64OC	Cantabrian Western	XY	W	Cangas de Narcea	Asturias
71OC	Cantabrian Western	XY	E	Somiedo	Asturias
77OC	Cantabrian Western	XY	E	Somiedo	Asturias
78OC	Cantabrian Western	XY	W	Somiedo	Asturias

Individual	Population	Sex	mtDNA Haplotype	Municipality (Recapture)	Province
80OC	Cantabrian Western	XX	W	Somiedo	Asturias
82OC	Cantabrian Western	XY	W	Teverga	Asturias
83OC	Cantabrian Western	XX	W	Teverga	Asturias
84OC	Cantabrian Western	XY	W	Cangas de Narcea	Asturias
85OC	Cantabrian Western	XY	W	Cangas de Narcea	Asturias
86OC	Cantabrian Western	XY	W	Cangas de Narcea	Asturias
87OC	Cantabrian Western	XY	W	Degaña	Asturias
90OC	Cantabrian Western	XY	W	Somiedo<br>(Somiedo)	Asturias
92OC	Cantabrian Western	XY	E	Somiedo	Asturias
93OC	Cantabrian Western	XY	E	Somiedo<br>(Somiedo)	Asturias
95OC	Cantabrian Western	XY	W	Belmonte de Miranda	Asturias
96OC	Cantabrian Western	XY	W	Belmonte de Miranda	Asturias
104OC	Cantabrian Western	XY	W	Somiedo	Asturias
107OC	Cantabrian Western	XY	W	Somiedo	Asturias
112OC	Cantabrian Western	XY	W	Somiedo	Asturias
113OC	Cantabrian Western	XY	W	Somiedo	Asturias
116OC	Cantabrian Western	XY	W	Somiedo	Asturias
126OC	Cantabrian Western	XY	W	Belmonte de Miranda	Asturias
128OC	Cantabrian Western	XY	W	Somiedo	Asturias
16OR	Cantabrian Eastern	XY	E	La Pernia	Palencia
18OR	Cantabrian Eastern	XY	E	Brañosera	Palencia
21OR	Cantabrian Eastern	XY	E	Cervera de Pisuerga<br>(Cervera de Pisuerga)	Palencia
23OR	Cantabrian Eastern	XY	E	Cabezon de Liebana	Cantabria
26OR	Cantabrian Eastern	XY	E	La Pernia	Palencia
28OR	Cantabrian Eastern	XY	E	La Pernia	Palencia
30OR	Cantabrian Eastern	XY	E	La Pernia	Palencia
31OR	Cantabrian Eastern	XY	E	La Pernia	Palencia
32OR	Cantabrian Eastern	XY	E	La Pernia	Palencia
33OR	Cantabrian Eastern	XY	E	A. de Campo/Valdeolea	Palencia
37OR	Cantabrian Eastern	XY	E	Cervera de Pisuerga	Palencia
38OR	Cantabrian Eastern	XY	E	La Pernia	Palencia
39OR	Cantabrian Eastern	XY	E	La Pernia	Palencia
40OR	Cantabrian Eastern	XY	E	La Pernia	Palencia
109OR	Cantabrian Eastern	XY	E	Velilla del Rio Carrion	Palencia
115OR	Cantabrian Eastern	XY	E	La Pernia	Palencia
118OR	Cantabrian Eastern	XY	E	Cervera de Pisuerga	Palencia
120OR	Cantabrian Eastern	XY	E	Prioro	Leon
124OR	Cantabrian Eastern	XY	E	Cervera de Pisuerga	Palencia
125OR	Cantabrian Eastern	XX	E	Cremenes	Leon
127OR	Cantabrian Eastern	XX	E	La Pernia	Palencia
1290R	Cantabrian Eastern	XY	E	Guardo	Palencia
131OR	Cantabrian Eastern	XX	E	Cremenes	Leon
132OR	Cantabrian Eastern	XX	E	Cervera de Pisuerga	Palencia
133OR	Cantabrian Eastern	XY	E	Cervera de Pisuerga	Palencia

Individual	Population	Sex	mtDNA Haplotype	Municipality (Recapture)	Province
136OR	Cantabrian Eastern	XY	E	Fresno del Rio	Palencia
137OR	Cantabrian Eastern	XY	E	Valdeolea	Cantabria
138OR	Cantabrian Eastern	XX	E	La Pernia	Palencia
140OR	Cantabrian Eastern	XY	E	Triollo	Palencia
142OR	Cantabrian Eastern	XY	E	La Pernia	Palencia
