# Supplementary material for: Paths for colonization or exodus? New insights from the brown bear (Ursus arctos) population of the Cantabrian Mountains
Source: PLoS One. 2020 Jan 31;15(1):e0227302. doi: 10.1371/journal.pone.0227302 (PMC6996475; doi:10.1371/journal.pone.0227302)
Supplement: S5 Table — (PDF) [file pone.0227302.s007.pdf]

SUPPORTING INFORMATION S6 - Estimation of migration rates and migrant identification using BAYESASS and GENECLASS.

Gregório, I, Barros, T, Pando, D, Morante, J, Fonseca, C, Ferreira, E (2019). A path for colonization or exodus? New insights from the Cantabrian brown bear population. PLOS One (submitted).
Eduardo Ferreira (Corresponding author, e-mail: elferreira@ua.pt). Department of Biology & CESAM, University of Aveiro, Campus Universitário de Santiago, 3810-193 Aveiro, Portugal.

Results:

Table S6.1 – Results from first-generation migrants detection using BAYESASS, GENECLASS and STRUCTURE + HYBRDLAB. Population refers to population in which each individual was sampled. **BAYESASS**: posterior probability of assignment of each individual to migrant and non-migrant classes. Highest posterior probability values for each individual are highlighted in grey. **GENECLASS**: individual likelihood ratios (L_{Home}/L_{Max}) of detection of first-generation markers, using Paetkau et al (1995) algorithm. Individuals identified as 1st generation migrants are highlighted in bold (algorithm without simulation for inference of statistical significance) and in bold underlined (algorithm with simulation). **STRUCTURE+HYBRIDLAB**: Assignment of individuals based on STRUCTURE results and thresholds inferred with HYBRIDLAB.

		**Posterior Probability of Assignment (BAYESASS)**			**L = L_{Home}/L_{Max} (GENECLASS)**	**Assignment (STRUCTURE + HYBRIDLAB)**
**Individual**	**Population**	Non-migrant	1st Gen. Migrant	2nd Gen. Migrant	Paetkau et al (1995)	
104OC	Cantabria Western	1.000			0.000	Not migrant
107OC	Cantabria Western	0.003	0.189	0.808	0.000	Admixed
10OC	Cantabria Western	0.999		0.001	0.000	Not migrant
112OC	Cantabria Western	0.134	0.425	0.441	0.000	Admixed
113OC	Cantabria Western	0.998		0.002	0.000	Not migrant
116OC	Cantabria Western	1.000			0.000	Not migrant
11OC	Cantabria Western	0.944		0.056	0.000	Not migrant
126OC	Cantabria Western		1.000		**3.706**	Admixed
128OC	Cantabria Western	0.999		0.001	0.000	Not migrant
12OC	Cantabria Western	0.999		0.001	0.000	Not migrant
14OC	Cantabria Western	0.000	0.998	0.002	1.775	1st Generation Migrant
15OC	Cantabria Western	0.998		0.002	0.000	Not migrant
1OC	Cantabria Western	1.000			0.000	Not migrant
2OC	Cantabria Western	1.000			0.000	Not migrant
3OC	Cantabria Western	0.987		0.013	0.000	Not migrant
44OC	Cantabria Western	0.974		0.025	0.000	Not migrant

Individual	Population	Posterior Probability of Assignment (BAYESASS)			$L = L_{Home}/L_{Max}$ (GENECLASS)	Assignment (STRUCTURE + HYBRIDLAB)
		Non-migrant	1st Gen. Migrant	2nd Gen. Migrant	Paetkau et al (1995)	
45OC	Cantabria Western	0.996		0.004	0.000	Not migrant
49OC	Cantabria Western	0.003	0.947	0.050	2.117	1st Generation Migrant
4OC	Cantabria Western	1.000			0.000	Not migrant
50OC	Cantabria Western	0.218		0.782	0.000	Admixed
51OC	Cantabria Western	1.000			0.000	Not migrant
52OC	Cantabria Western	1.000			0.000	Not migrant
53OC	Cantabria Western	0.986		0.014	0.000	Not migrant
55OC	Cantabria Western	0.999		0.001	0.000	Not migrant
57OC	Cantabria Western	0.999		0.001	0.000	Not migrant
59OC	Cantabria Western	0.997		0.003	0.000	Not migrant
60OC	Cantabria Western	0.994		0.006	0.000	Not migrant
61OC	Cantabria Western	0.988		0.012	0.000	Not migrant
62OC	Cantabria Western	1.000			0.000	Not migrant
63OC	Cantabria Western	0.999		0.001	0.000	Not migrant
64OC	Cantabria Western	0.994		0.006	0.000	Not migrant
6OC	Cantabria Western	1.000			0.000	Not migrant
71OC	Cantabria Western		0.995	0.005	2.587	1st Generation Migrant
77OC	Cantabria Western		0.999	0.001	**2.988**	1st Generation Migrant
78OC	Cantabria Western	0.807		0.193	0.000	Not migrant
7OC	Cantabria Western	0.999		0.001	0.000	Not migrant
80OC	Cantabria Western	0.999		0.001	0.000	Not migrant
82OC	Cantabria Western	0.219		0.781	0.000	Admixed
83OC	Cantabria Western	0.997		0.003	0.000	Not migrant
84OC	Cantabria Western	0.682		0.318	0.000	Not migrant
85OC	Cantabria Western	0.990		0.010	0.000	Admixed
86OC	Cantabria Western	1.000			0.000	Not migrant
87OC	Cantabria Western	0.996		0.004	0.000	Not migrant
8OC	Cantabria Western		0.985	0.015	**6.035**	1st Generation Migrant
90OC	Cantabria Western	1.000			0.000	Not migrant
92OC	Cantabria Western		0.998	0.002	**3.766**	1st Generation Migrant
93OC	Cantabria Western		0.916	0.084	2.261	1st Generation Migrant
95OC	Cantabria Western	1.000			0.000	Not migrant

		Posterior Probability of Assignment (BAYESASS)			$L = L_{Home}/L_{Max}$ (GENECLASS)	Assignment (STRUCTURE + HYBRIDLAB)
**Individual**	**Population**	Non-migrant	1st Gen. Migrant	2nd Gen. Migrant	Paetkau et al (1995)	
96OC	Cantabria Western	0.999		0.001	0.000	Not migrant
9OC	Cantabria Western	1.000			0.000	Not migrant
109OR	Cantabria Eastern	0.989		0.011	0.000	Not migrant
115OR	Cantabria Eastern	0.997		0.003	0.000	Not migrant
118OR	Cantabria Eastern	0.962		0.038	0.000	Admixed
120OR	Cantabria Eastern	0.998		0.002	0.000	Not migrant
124OR	Cantabria Eastern	0.997		0.003	0.000	Not migrant
125OR	Cantabria Eastern	1.000			0.000	Not migrant
127OR	Cantabria Eastern	1.000			0.000	Not migrant
129OR	Cantabria Eastern	0.999		0.001	0.000	Not migrant
131OR	Cantabria Eastern	1.000			0.000	Not migrant
132OR	Cantabria Eastern	1.000			0.000	Not migrant
133OR	Cantabria Eastern	0.998		0.002	0.000	Admixed
136OR	Cantabria Eastern	0.999		0.001	0.000	Not migrant
137OR	Cantabria Eastern	1.000			0.000	Not migrant
138OR	Cantabria Eastern	1.000			0.000	Not migrant
140OR	Cantabria Eastern	1.000			0.000	Not migrant
142OR	Cantabria Eastern	0.991		0.009	0.000	Not migrant
16OR	Cantabria Eastern	1.000			0.000	Not migrant
18OR	Cantabria Eastern	1.000			0.000	Not migrant
21OR	Cantabria Eastern	0.967		0.032	0.000	Admixed
23OR	Cantabria Eastern	0.884	0.001	0.116	0.320	Admixed
26OR	Cantabria Eastern	1.000			0.000	Not migrant
28OR	Cantabria Eastern	1.000			0.000	Not migrant
30OR	Cantabria Eastern	0.997		0.003	0.000	Not migrant
31OR	Cantabria Eastern	1.000			0.000	Not migrant
32OR	Cantabria Eastern	1.000			0.000	Not migrant
33OR	Cantabria Eastern	1.000			0.000	Not migrant
37OR	Cantabria Eastern	0.998		0.002	0.000	Not migrant
38OR	Cantabria Eastern	1.000			0.000	Not migrant
39OR	Cantabria Eastern	1.000			0.000	Not migrant
40OR	Cantabria Eastern	0.913	0.025	0.061	**2.184**	Admixed

Table S6.2 - Migration Rates among Cantabria Western and Eastern populations (with standard deviation), inferred using BAYESASS.

To<br>From	Cantabria Western	Cantabria Eastern
**Cantabria Western**	0.916 ± 0.0211	0.014 ± 0.0128
**Cantabria Eastern**	0.084 ± 0.0211	0.986 ± 0.0128

Table S6.3 - Migration Rates among Cantabria Western and Eastern populations (with 95% CI), inferred using BAYESASS. * - migration rate was significantly higher from Cantabria Eastern to Western population.

To<br>From	Cantabria Western	Cantabria Eastern
**Cantabria Western**	0.916 ± 0.0414	0.014 ± 0.0251*
**Cantabria Eastern**	0.084 ± 0.0414*	0.986 ± 0.0251

Table S6.4 - Inbreeding Coefficients for Cantabria Western and Eastern populations, inferred using BAYESASS. The inbreeding coefficient was significantly higher (and different from 0) in the Cantabria Eastern population.

Subpopulation	Inbreeding Coefficient	Standard deviation	95% Confidence Intervals
**Cantabria Western**:	0.0170	± 0.0143	± 0.0280
**Cantabria Eastern**:	0.1066*	± 0.0332	± 0.0650
