# Supplementary material for: Paths for colonization or exodus? New insights from the brown bear (Ursus arctos) population of the Cantabrian Mountains
Source: PLoS One. 2020 Jan 31;15(1):e0227302. doi: 10.1371/journal.pone.0227302 (PMC6996475; doi:10.1371/journal.pone.0227302)
Supplement: S6 Table — (PDF) [file pone.0227302.s008.pdf]

SUPPORTING INFORMATION S8 - Allele frequencies per loci, for the entire Cantabrian population, the Eastern and Western subpopulations.

Gregório, I, Barros, T, Pando, D, Morante, J, Fonseca, C, Ferreira, E (2019). A path for colonization or exodus? New insights from the Cantabrian brown bear population. PLOS One (submitted).

Eduardo Ferreira (Corresponding author, e-mail: elferreira@ua.pt). Department of Biology & CESAM, University of Aveiro, Campus Universitário de Santiago, 3810-193 Aveiro, Portugal.

Table S8. Allele frequencies per locus for the entire Cantabrian population, the Eastern and Western Cantabrian subpopulations, as well as the Western Cantabrian subpopulation without migrants. Private alleles in each subpopulation are underlined. Private alleles were the same for the Eastern Cantabrian subpopulation whether it was compared with the Western Cantabrian subpopulation with or without migrants.

**Locus**		**Cantabria**	**Western**	**Western without migrants**	**Eastern**
	Sample N	*80*	*50*	*43*	*30*
	**Alleles**				
**MU50**	**93**	0.109	0.167	0.143	0.052
	**95**	0.487	0.488	0.480	0.500
	**99**	0.295	0.143	0.204	0.448
	**103**	0.109	0.202	0.173	
**MU23**					
	**143**	0.444	0.267	0.290	0.700
	**145**	0.275	0.349	0.370	0.117
	**151**	0.200	0.372	0.320	
	**154**	0.081	0.012	0.020	0.183
**MU59**					
	**85**	0.006			0.018
	**92**	0.338	0.060	0.122	0.714
	**94**	0.364	0.667	0.571	
	**96**	0.162	0.048	0.102	0.268
	**108**	0.130	0.226	0.204	
**G10L**					
	**145**	0.064	0.116	0.100	
	**152**	0.032	0.058	0.050	
	**156**	0.532	0.616	0.590	0.429

	**158**	0.372	0.209	0.260	0.571
**G10P**					
	**127**	0.487	0.683	0.583	0.328
	**136**	0.006		0.010	
	**143**	0.357	0.280	0.281	0.483
	**145**	0.149	0.037	0.125	0.190
**G10J**					
	**54**	0.007			0.017
	**57**	0.408	0.263	0.298	0.586
	**65**	0.408	0.400	0.415	0.397
	**75**	0.178	0.338	0.287	
**G1A**					
	**108**	0.013			0.033
	**114**	0.436	0.537	0.469	0.383
	**116**	0.551	0.463	0.531	0.583
**MU61**					
	**141**	0.167	0.275	0.234	0.040
	**143**	0.833	0.725	0.766	0.960
**MU51**					
	**90**	0.033	0.012	0.010	0.077
	**124**	0.020	0.035	0.030	
	**127**	0.204	0.209	0.180	0.250
	**129**	0.743	0.744	0.780	0.673
**G10X**					
	**121**	0.128	0.131	0.115	0.150
	**126**	0.571	0.798	0.729	0.317
	**128**	0.231	0.060	0.073	0.483
	**130**	0.071	0.012	0.083	0.050
**G1D**					
	**171**	0.032	0.061	0.052	
	**177**	0.968	0.939	0.948	1.000
**MU05**					
	**125**	0.241	0.167	0.163	0.367
	**127**	0.291	0.262	0.276	0.317
	**129**	0.057	0.024	0.031	0.100
	**143**	0.411	0.548	0.531	0.217
**G10C**					
	**100**	0.500	0.709	0.670	0.196
	**103**	0.500	0.291	0.330	0.804
**MU10**					

	**117**	0.164	0.122	0.125	0.232
	**129**	0.079	0.024	0.063	0.107
	**131**	0.757	0.854	0.813	0.661
**MU09**					
	**181**	0.279	0.405	0.354	0.155
	**200**	0.156	0.071	0.125	0.207
	**203**	0.299	0.321	0.281	0.328
	**209**	0.266	0.202	0.240	0.310
